# Pulse Knowledge, Attitudes, Practices, and Cooking Experience of Midwestern US University Students

**DOI:** 10.3390/nu12113499

**Published:** 2020-11-13

**Authors:** Donna M. Winham, Elizabeth D. Davitt, Michelle M. Heer, Mack C. Shelley

**Affiliations:** 1 Department of Food Science & Human Nutrition, Iowa State University, Ames, IA 50010, USA; eddavitt23@gmail.com (E.D.D.); mmheer@gmail.com (M.M.H.); 2 Departments of Political Science and Statistics, Iowa State University, Ames, IA 50010, USA; mshelley@iastate.edu

**Keywords:** college students, pulses, legumes, beans, young adults, cooking

## Abstract

Many American college students fail to meet dietary guideline recommendations for fruits, vegetables, and fiber. Pulses are a subgroup of legumes, harvested solely for dry grain seeds within a pod. Commonly consumed pulses include dry beans, dry peas, lentils, and chickpeas. Pulses are high in shortfall nutrients and could fill some nutritional gaps of college students. However, little is known about pulse intakes among young adults. The study aims were: (1) to identify knowledge, attitudes, and practices regarding pulse consumption; and (2) to describe experiences of preparing dry pulses among college students. A convenience sample of 1433 students aged 18–30 enrolled at a Midwestern university in the United States completed an online survey in April 2020. Demographic and attitude variables were compared by the monthly count of pulse types eaten using chi-square, analysis of variance, and logistic regression modeling to predict pulse type intakes. Higher numbers of pulse types eaten was associated with being White, vegetarian/vegan, higher cooking self-efficacy, positive attitudes toward pulses, and greater daily intake of fruits, vegetables, and fiber. Knowledge and experience of cooking dry pulses was low, with canned pulses purchased more often. College students may not be consuming pulses due to unfamiliarity with them, low knowledge of nutrition benefits, and a general lack of cooking self-efficacy. Increased familiarization and promotion surrounding pulses may increase their consumption.

## 1. Introduction

Young adults, as with most Americans, do not meet recommended dietary intakes for fruits, vegetables, and fiber [1]. From adolescence into adulthood, fruit and vegetable consumption tend to decline [2]. The transition from adolescence to emerging adulthood is a critical time to establish eating behaviors that will shape an individual’s health for a lifetime [2,3]. College students are no exception to this pattern. The university setting, with its structured meal plans and housing, limited food outlets, and time constraints, may shape behavior and restrict food choice. Largely cited reasons of college students for not consuming healthier food options are time limitations and lack of food preparation knowledge [4]. In a qualitative investigation, college students justified unhealthy eating practices as part of a “typical college lifestyle” with little they can do to change [4]. Thus, college students may be nutritionally vulnerable, lacking in certain nutrients, particularly fiber, folate, and iron, and at risk of weight gain [2,5]. Based on United States (US) national college data from spring 2020, only 18% met fruit and 32% met vegetable recommendations of three or more servings per day [6]. For busy college students, canned fruits and vegetables are a convenient and inexpensive way to increase dietary quality [7]. However, misconceptions and lack of awareness about canned products may detract from their use [8]. Foods that can fill nutritional gaps for college students, as well as meet their food preferences of easy preparation and storage and low cost, could improve the dietary quality of these young adults [7,8]. One such food type to meet these criteria are pulses, which come in a range of flavors, textures, and colors [9].

Pulses, as defined by the United Nations Food and Agriculture Organization (FAO), are annual leguminous crops yielding grain seeds within a pod. The definition is “limited to crops harvested solely for dry grain, thereby excluding crops harvested fresh for food (green peas, green beans, etc.) which are classified as vegetable crops” [10]. Oilseeds (e.g., soybeans and peanuts) and field cover crops (e.g., clover and alfalfa) are not pulses [10]. Although there are 11 primary pulse crops globally, in the US dry beans, dry peas, lentils, and chickpeas are the most frequently consumed types [10,11]. Dry beans have many different market classes such as pinto, black, navy, and kidney. Beans are the best-known pulse type in the US, but all pulses share low-fat, high-protein, high-fiber characteristics, and many positive health benefits [12,13].

The 2015–2020 Dietary Guidelines for Americans (DGA) uses the term “legumes” but it is more similar to that of the FAO term pulses, due to similar exclusion of fresh peas and green (string) beans [1]. Unlike FAO, the DGA allows classification of legumes as either a protein or vegetable because of their diverse nutrient profile [1,10]. Most pulses are high in fiber, folate, iron, and potassium, which are shortfall nutrients in the US [11,12,13]. The DGA advocates for 1.5 cups per week of dry, mature legumes for adults [1]. Pulse consumption of 0.5 cup daily has been associated with weight management, and lower risks of chronic conditions, including type 2 diabetes and cardiovascular disease [9,11,12,13]. Since most young adults are healthy at this age, nutritional risk related to chronic disease development is a poor motivator for healthy eating [2,3].

Some studies have demonstrated positive attitudes among college students for sustainable production practices, consumption of plant-based diets, and vegetarianism [14,15]. Notably, pulses contribute to sustainable food systems through their ability to fix nitrogen, reduce fertilizer usage, and improve soils [16]. Compared to animal-based proteins, all legumes have a lower effect on greenhouse gas emissions, energy use, and other environmental damage [17]. Despite their sustainability advantage, clear health benefits, versatility, easy preparation (especially with canned varieties), and affordability, pulses are consumed at levels below recommendations across most age groups [1,11,12,13]. Research on college students in a southeastern US university and a Canadian university both showed pulse consumption below the dietary recommendations for the majority of students [18,19]. Establishing healthy diet patterns that include the recommended amount of pulses may improve nutrition and address global environmental challenges [16,17].

Research is limited on specific foods purchased and the attitudes or purchasing preferences of college students or other young adults (aged 18–30) [2,3,4,7,18,19,20]. Few studies have assessed US college students’ knowledge, attitudes, and practices surrounding pulse usage or factors influencing pulse consumption. The objectives of this cross-sectional study were: (1) to identify knowledge of the term “pulses”, attitudes, and practices regarding pulse consumption; and (2) to describe experiences of preparing dry pulses. This information can guide future intervention messaging about consuming and cooking pulses.

## 2. Materials and Methods

### 2.1. Study Design and Participants

A convenience sample of enrolled students aged 18–30 attending Iowa State University in the Midwestern US completed an online survey in spring 2020. The overarching study design and eligibility criteria were based on evaluating variables of food security and dietary intakes at the onset of the novel coronavirus (COVID-19) pandemic. The current analysis on pulse consumption is from a subset of these questions. Enrolled students who did not live in Ames, Iowa, as of March 2020 were ineligible (e.g., study abroad, internship, and online only). Professional students in the College of Veterinary Medicine were excluded because they differ demographically from other students (older, more likely married, not on the main campus). With administration permission, one survey invitation was sent to 29,810 university email addresses between 26–30 April 2020, using an online survey platform (Advantage Plan 2020, Survey Monkey, San Mateo, CA, USA). Of these, 12,958 were not opened, 19,152 were known to be received (2113 clicked on the link, 17,039 opened), and 1907 survey responses were started. The response rate was ~10% (1907/19,152). The subject lines of emails mentioned influences on diet, food shopping, and post-COVID experiences, but not pulses to reduce potential respondent bias.

The study invitation stated that students would receive a $5 e-gift card to Amazon after response checks for plausibility and if the survey was at least 75% complete. The total survey consisted of 67 possible questions depending on skip patterns. Fourteen questions required a response to advance. These compulsory questions consisted of six for eligibility, two integrity checks [21], and six yes/no items that governed skip patterns to continue in the survey. All other questions were optional. The survey was pilot-tested with 17 college students, and five nutrition faculty reviewed the survey content. Minor changes were made in wording and flow based on feedback. Respondents spent an average of 17 minutes on the survey. Iowa State University’s Institutional Review Board approved the study (#16-289). Survey completion was considered informed consent.

### 2.2. Survey Development

The survey included demographic, self-reported health risk, and vegetarian/vegan diet questions used in previous validated research [20,22]. A food frequency screener for fruit, vegetable, and fiber intakes provided data on consumption of 10 food groups [23]. Estimates of the number of fruit and vegetable servings and grams of fiber consumed by age and sex were generated by entering responses into the NutritionQuest validated online screener [23,24]. Seven of 12 validated general cooking self-efficacy questions were summed to create a score [7]. Responses were provided on a 4-point Likert scale (not at all confident, a little confident, somewhat confident, very confident) for ability to follow recipe directions, make a salad, prepare a stir-fry, make a casserole, create baked goods, and produce a hot meal [7]. Reliability analysis indicated a “good” Cronbach’s alpha (0.79) for the scale. Food security was measured using the six-item U.S. Household Food Security Survey Module [25].

Respondents were asked to select the best definition of the terms “legumes” and “pulses” from multiple options [26]. Wording for the correct definitions were based on common terminology used by the North American pulse groups and as utilized in a previous study of registered dietitians [11,12,26]. Participants were provided a definition of pulses and examples before they were asked further questions about pulses. Purchasing pattern questions asked if they routinely bought dry beans, peas, lentils, chickpeas, or other pulses that needed to be cooked, and if they purchased canned beans such as baked beans, pinto, black, and other [27,28]. Participants self-reported if they ate 11 different popular pulse types in the US, pulse-based pastas, and snack foods. Response categories were eat often (defined as once per month or more), have eaten, never eaten, and do not know) [11,29]. Behavior questions on the purchase of canned and dry beans and cooking of dry pulses were asked for the previous 30 days at the time of data collection.

Likert-type attitude statements on canned foods, high-fiber foods causing gas, dry pulse preparation, and bean taste were used from previous validated research [27,28]. Pulse cooking experience questions were used from the Global Pulse Confederation 2020 “Cooking times: Citizen Science” survey [30]. Questions on behavioral responses to COVID-19, plant-based alternatives to meat, and ecological attitudes are not reported here.

### 2.3. Data Transformations and Analysis

All data analysis was performed using SPSS v26. (IBM, Armonk, NY, USA). The food security summary score was recoded into high, low, and very low categories per the instrument instructions [25]. Likert-type attitude statement responses of strongly disagree, disagree, neutral, agree, and strongly agree were condensed to disagree, neutral, and agree for clarity in tables. The responses of “eat often” (defined as once a month or more) for 11 pulse types were summed. The total pulse type count was not normally distributed. Based on the frequency distribution, it was recoded into three categories (none, 1–2 types eaten, and 3–11 types eaten). Categories represent the count of pulse types eaten per month. Demographics, pulse knowledge, attitudes, cooking self-efficacy, and dry pulse cooking experiences were compared by the three pulse type count groups using chi-square and analysis of variance (ANOVA). Those variables that were significant by pulse type count or theoretically important, e.g., gender and food security status, were entered into logistic regression analysis to predict bivariate pulse consumption (none, 1+ per month).

## 3. Results

Of the 1907 survey responses, 165 participants did not meet age, location, or other eligibility criteria in the initial screening questions and disqualified from progressing further in the survey. Another 271 respondents had incomplete variables of interest for this analysis, and 38 failed two integrity items that checked for seriousness of responses (level of honesty in answering questions and the degree to which the answers are accurate). The analysis sample of 1433 was 61% female and 82% White, with a mean age of 21.4 ± 2.7 years. Fifty-six percent were from Iowa, 35% from other US states, and 9% international students. In comparison, spring 2020 University enrollment data indicated the overall population was 43% female, 85% White, 60% from Iowa, 35% from other US states, and 5% international students [31].

**Figure 1:**
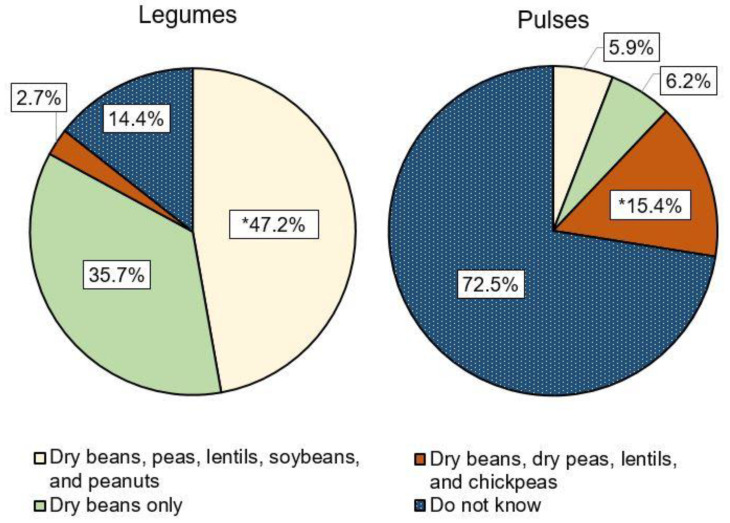
Response percentages for knowledge of the terms “legumes” and “pulses” by students enrolled at a Midwestern US university (*n* = 1433); * correct answer.

Knowledge of the correct definition for “legumes” was greater than for “pulses” (47.2% vs. 15.4%). Only 14.4% of respondents reported they did not know the definition of a legume, whereas 72.5% did not know the term pulse (Figure 1).

The pulses and pulse food items students reported to “eat often” or at least once per month include chili made with beans, 36%; chickpeas (hummus, on salads, and dahls), 30%; pulse-based snacks (puffs, chips, etc.), 28%; baked beans/pork and beans, 27%; peas (in casseroles and split pea soup), 26%; and refried beans (side, tacos, and burritos), 26% (Table 1). As described in the Methods section, the responses of “eat often” for 11 pulses were transformed into an ordinal variable representing the number of different pulse types eaten per month for analysis. Thirty-two percent of students reported not eating any pulses per month, 31% reported 1–2 types eaten per month, and 37% indicated they ate 3–11 of the pulse types on a monthly basis.

Demographics and diet characteristics by number of pulse types eaten categories are shown in Table 2. Females, Whites, vegetarians/vegans, and those with very good to excellent self-reported health status ate significantly greater numbers of pulse types. Those who ate more pulse types had significantly higher intakes of five or more servings of fruits and vegetables per day and more than 20 g of fiber per day. The cooking self-efficacy score was normally distributed and significantly higher among those who ate more pulse types.

When asked about the form of pulses routinely purchased, 7% bought dry only, 28% purchased canned pulses only, 22% purchased both dry and canned forms, and 43% did not buy any. There was a significant difference between pulse non-consumers and consumers regarding their association of canned foods with poor people, but, overall, 94% disagreed with this statement. Those who consumed a greater number of pulse types disagreed with statements that dry beans took too long to prepare from scratch and that they did not like the taste of beans (both *p* < 0.001) (Table 3).

Table 4 shows pulse-cooking experiences of students by pulse type count groups. As the pulse type count increases, significantly more respondents indicate knowing how to cook them. Overall, 63% stated that they do not know how to prepare dry beans and other pulses, and 64% stated that they had never tried to cook pulses. Of the 36% who had cooked dry pulses, only 12% said that it was their first attempt in the past 30 days. The most recent pulse types cooked were black beans, lentils, pinto beans, and chickpeas. Sixty-six percent said that they soaked pulses prior to cooking, 66% used a pot on a stove, 17% used a pressure cooker, and 9% used a slow cooker. Eighty-seven percent of those who tried to cook dry pulses reported a positive experience. Of the 13% of respondents who had a negative experience when cooking pulses, about 31% did not give any reason other than they did not like the taste of the particular pulse cooked. For the other 69%, the main reasons given related to improper preparation (27% did not soak them long enough, 28% did not cook enough, 25% lacked instructions on how to cook, 8% did not rinse well, 5% cooked too long, and 3% pulses were old; multiple responses could be selected; data not shown).

Gender, race, vegetarian/vegan status, cooking self-efficacy, food security, and three attitude statements were entered into a logistic regression model to predict pulse type count frequency (0 pulses; 1+ pulse types). The most parsimonious model included five variables (cooking self-efficacy score, vegetarian/vegan, race, and two pulse attitude statements; Table 5). The model correctly predicts 73.2% of pulse consumption, including 37.8% of instances for no pulses eaten and 89.4% instances of pulses eaten at least once monthly. All included variables are statistically significant predictors. Fit metrics for logistic regression indicate that the predictive model performs adequately (Nagelkerke pseudo-*R* (coefficient of determination, measuring explained variation)^2^ = 0.217 and Cox and Snell pseudo-*R*^2^ = 0.154).

## 4. Discussion

College-aged students experience many life changes, and unhealthy diets have become commonplace [2,4]. Pulses are foods that can fill nutritional gaps for college students (fiber, folate, and iron), as well as meet their food preferences and improve dietary quality of these young adults [1,6,18,19]. Assessing current consumer practices is the first step in developing an evidence-based plan to do so.

The aim of this cross-sectional study was to address the absence of information on pulse consumption among college-aged adults at a Midwestern university in the US. The first objective was to identify college students’ knowledge, attitudes, and practices regarding pulse consumption, such as preferred form (canned or dry), type of pulse, or pulse products eaten. The second objective was to describe experiences of college students preparing dry pulses to gain information for future messaging about cooking pulses. Key findings show college students had limited knowledge about the term “pulses” and estimated consumption below recommended intakes, but overall positive attitudes. Knowledge and experience of cooking dry pulses was low but an enjoyable experience for most who had tried this activity.

From the logistic regression model, those who were more likely to consume pulses at least once per month were White, vegetarian/vegan, had higher cooking self-efficacy, and had positive attitudes toward canned foods and beans. These results are consistent with other data showing that those who are vegetarian or live in a household with a vegetarian are more likely to consume legumes [32,33]. Higher pulse type counts were associated with better health status, and greater daily intake of fruits, vegetables, and fiber. Cooking skills have been correlated to increased fruit and vegetable consumption and more home-cooked meals [34]. Analysis of the National Health and Nutrition Examination Survey 1999–2002 data shows bean consumers have a higher intake of dietary fiber compared to those who do not consume beans [35]. As with the college students in this study, other research supports that most people have a positive attitude toward beans/pulses, like the taste of them, and purchase both dry and canned [18,28,36,37,38,39]. Black beans, lentils, pinto beans, and chickpeas were the most common types of pulses cooked, which is similar to pulse popularity found in other studies in North America [11,18,19,28,36].

The perception of high-fiber foods such as pulses causing intestinal gas or discomfort after eating is a barrier cited in other studies [39,40]. Although a slight majority of the Midwestern college sample agreed that high fiber foods can cause gas, there was no difference by pulse type count groups. Whether or not intestinal gas was a barrier specifically for pulse consumption was not asked in this study. The preoccupation with pulses and gas may be somewhat exaggerated, and transitory at best [41]. Other studies suggest the public is unaware of positive intestinal health benefits from pulses or high fiber foods [37,42].

Young adults cite barriers such as a lack of time to prepare foods and a lack of skills as reasons for not cooking at home [4,34,43,44]. Canned foods generally provide a quick and easy way to prepare a nutritious meal and may provide a mechanism to increase pulse consumption in students with lower cooking self-efficacy [7,8,45]. US consumers often prefer canned options due to convenience [45]. Canned foods are low-cost, have longer storage potential, and are a nutrient-dense option [45]. New pulse varieties and shorter cooking and canning times are improving nutrition quality further [46]. However, the college students disagreed that canned foods are as nutritious as frozen or fresh suggesting a need for further education [7,8]. Frequent canned food consumption is correlated with higher intakes of nutrient dense foods and overall healthier eating habits [45]. The DGA 2015–2020 states that “All forms of foods, including fresh, canned, dried, and frozen, can be included in healthy eating patterns” [1]. One study found that having college students try three recipes using canned vegetables improved their perceptions of the nutrient content, contributions to the DGA, and ultimately increased their usage of canned vegetables [7].

Students were more knowledgeable about the term “legume” than “pulse”. Other studies with college students have shown a deficit of pulse knowledge [7,18,19]. Only 36% of college students were able to identify beans or legumes correctly from a list of 10 food items [18]. Another study found that college students had confusion about the definition of “dry legumes (beans)” during the development of a questionnaire regarding factors related to the use of canned foods [7].

The observed frequency of 68% of students consuming 1–2 pulse types per month is consistent with some other studies. A southeastern university sample of 355 college students found that 67% consumed beans at least 1–3 times a month [18]. However, the amount of beans reported was only 0.75 of a cup per week, which is far less than the DGA recommendation of 1.5 cups per week [1,16]. Even lower intakes were observed in a Canadian study, where only about 50% consumed them 1–3 times per month [19]. A Spanish study with first-year university students cited daily bean consumption of less than 0.25 cup per day [47]. Similar research conducted with participants of an older age and greater ethnic/racial diversity than the Midwestern US college students showed higher pulse consumption rates. Eighty percent of Hispanic and Non-Hispanic White women in Iowa [28,42], 89% of Hispanic and Non-Hispanic White women in Arizona [36], and 79% of low-income White, Black, and Hispanic men in Arizona [37] reported consuming beans at least 2–3 times per month. Just over half of adults ≥65 years old in a Canadian study reported consuming beans on a daily or weekly basis [39].

The second focus of this study was to understand factors related to college students’ experiences with cooking pulses. Higher pulse consumption among the Midwestern university students was associated with knowing how to prepare them, experience cooking dry pulses, and reporting a positive experience cooking pulses. Other studies have suggested that the most common reasons for not consuming pulses are general dislike or dislike of taste; a lack of familiarity, knowledge of recipes, and knowledge of preparation; and low interest [32,40,48,49]. Having the ability to prepare bean meals and considering them as tasty was associated with increased likelihood of bean consumption [50]. Although most Midwestern US students did not know how to prepare pulses, of the students who had cooked dry pulses, the majority stated their experience was positive. The top two dry pulse cooking methods reported by students (pot on a stove and pressure cooker) were the same methods identified from preliminary analysis of a global study collecting information regarding pulse cooking procedures [30,51]. The most noted negative experiences of students with cooking pulses clustered around their lack of dry pulse preparation knowledge.

Limited cooking experience alongside pulse type consumption counts that are higher than purchasing frequencies suggests pulses are eaten in locations outside of the home or as ready-prepared items. An obstacle to pulse consumption seems to be related to self-preparation in this research and others [34]. Participants in a French study selected pulses to fit into restaurant and self-service scenarios more often than everyday situations [49]. The French participants were knowledgeable about the benefits of pulses but had low consumption and considered pulses “difficult to prepare” with the exception of lentils which were the least well liked or consumed [49]. Conversely, in the Midwestern college student survey, lentils, a quick cooking variety, were the second most frequently prepared pulse after black beans suggesting they are liked by this sample.

Knowledge of dry pulse preparation for these college students in the Midwestern US was lower compared to some other US studies. Eighty percent of low-income men and 90% of low-income women in Arizona [36,37] reported knowing know how to prepare dry beans. Florida participants in the Special Supplemental Nutrition Program for Women, Infants, and Children (WIC) were confident in their ability to cook dry beans [38]. Most adults in an Australian study responded that they knew how to cook legumes and found them easy to prepare [50]. Preparation knowledge may differ due to the older age or greater racial/ethnic diversity of samples in previous studies compared to the college student sample in this study. These differences highlight the necessity of identifying cultural and situational norms for pulse consumption [28,36].

This research is one the first studies to document pulse preparation experiences and adds to the limited literature on pulse knowledge and consumption of college students. These findings can inform intervention strategies for increasing pulse use among this vulnerable young adult cohort due to nutritional gaps of low fruit, vegetable, and fiber intakes, and high rates of food insecurity.

There are some limitations to the study. Overall legume intake frequency and serving size was not asked of participants, which limits direct comparison to DGA recommendations. Pulse availability in the college food environment including offerings at campus dining locations and popular off-campus food establishments was not conducted. Information on pulses served at university dining halls, obtained at restaurants, consumed as self-prepared or ready-made foods, or served by friends, family, or other meal programs was not assessed. Emerging dietary changes during the novel coronavirus (COVID-19) pandemic may have confounded dietary reports. Behavior questions were asked in the context of past 30 days/4 weeks. Data were collected through a convenience sample and thus are not representative of the university population.

## 5. Conclusions

This research has built upon limited data on pulse consumption, attitude, preferences, and cooking experience of college students. However, more research to test interventions for increasing pulse consumption is needed. Generally, the college student population may not be consuming pulses due to a lack of cooking self-efficacy, low knowledge of pulse preparation, and unfamiliarity with the foods. Methods could include the promotion of familiar items, such as bean chili, hummus, and pulse-based snacks, as well as recipes utilizing canned beans. Other strategies include taste testing and incorporation of pulses into cooking and nutrition classes offered to college students. As there are higher rates of pulse type consumption than pulse cooking, interventions targeted to increase and highlight pulse-based offerings through campus dining outlets, community food outlets, and pulse-based convenience foods may be consistent with students’ current pulse preferences. Disseminating information on the definition of pulses, benefits of canned pulses, and dry pulse preparation using students’ preference of lentils, black beans, pinto beans, and chickpeas can address knowledge deficits.

Future studies should further assess ways to improve college student nutrition, pulse familiarity, and pulse-cooking experiences. Additionally, research should investigate effective platforms for disseminating important pulse information and conducting interventions, such as hands-on cooking classes, social media, and throughout the university campus environment such as dining centers, food outlets, student housing, recreation centers, and student wellness.

## Figures and Tables

**Table 1:** Consumption frequencies of pulses and pulse food items of students enrolled at a Midwestern US university by (%; *n* = 1433).

Have You Ever Eaten or Do You Eat Often? ^a^	Eat Often	Have Eaten	Never Eaten
	%		
Chili made with beans	35.3	54.5	9.8
Chickpeas, hummus, on salads, dahls	30.3	49.3	20.0
Baked beans, or pork and beans	26.6	60.2	13.1
Peas, in casseroles, or split pea soup	26.5	60.4	13.1
Refried beans as side, in tacos, burritos	25.6	61.6	12.7
Black beans, or in salads, or mix	20.7	47.6	31.7
Kidney beans as side, on salads, in mix	20.3	60.4	19.3
Red beans, red beans, and rice	13.9	52.5	33.7
Lentils, in soup, mixes	13.7	53.9	32.4
Mung beans, sprouts	3.9	21.3	74.7
Black eyed peas, cowpeas	3.8	33.4	62.8
Pasta made with chickpeas or other pulses	10.2	39.7	50.1
Pulse-based snack puffs, chips, etc.	28.1	51.8	20.1

^a^ “Often” is defined as once a month or more.

**Table 2:** Demographic and health characteristics of students enrolled at a Midwestern US university by trivariate pulse type count eaten per month (%; *n* = 1433).

Characteristics	Total	No Pulses Eaten 32% (452)	1–2 Types Eaten 31% (451)	3–11 Types Eaten 37% (530)	*p*-Value
Cooking self-efficacy x ± SD	23.2 ± 4.2	22.1 ± 4.7	23.1 ± 4.0	24.3 ± 3.7	<0.001
	%				
Gender Male Female	38.8 61.2	40.7 59.3	42.4 57.6	34.2 65.8	0.019
Race White Other	82.0 18.0	79.2 20.8	80.9 19.1	85.3 14.8	0.037
Residency Status Iowa Other US State International	56.0 35.0 9.0	56.6 32.1 11.3	56.1 35.9 8.0	55.3 36.8 7.9	0.232
Vegetarian/vegan Yes No	8.6 91.4	4.2 95.8	6.2 93.8	14.3 85.7	<0.001
Food Security Status High Low Very low	82.6 11.4 6.1	80.1 12.8 7.1	82.0 12.4 5.5	85.1 9.2 5.7	0.262
Health Status Poor-Fair Good Very good-excellent	12.3 42.0 45.7	13.6 47.6 38.9	13.6 41.3 45.1	10.2 37.8 52.0	0.001
Servings of F/V per day <5 per day 5 or more per day	86.5 13.5	91.6 8.4	87.4 12.6	81.3 18.7	<0.001
Fiber grams per day <20 g of fiber per day 20+ g of fiber per day	66.6 33.4	81.6 18.4	66.3 33.7	54.0 46.0	<0.001

**Table 3:** Pulse purchasing patterns and attitudes of students enrolled at a Midwestern US university by trivariate pulse type count eaten per month (%; *n* = 1433).

Pulse Purchasing Preference and Attitude Statements	Total	No Pulses Eaten 32% (452)	1–2 Types Eaten 31% (451)	3–11 Types Eaten 37% (530)	*p*-Value
	%				
Pulse type purchased Both (dry and canned) Dry pulse only Canned pulse only Did not buy pulses	22.4 6.8 27.7 43.1	11.3 6.2 17.0 65.5	20.4 6.7 28.6 44.3	33.6 7.5 36.0 22.8	<0.001
**Attitude statements**					
Only poor people eat canned foods Disagree Neutral Agree	94.4 4.2 1.4	92.5 4.8 2.6	94.0 5.1 0.9	96.2 3.0 0.8	0.028
Canned foods are as nutritious as frozen or fresh Disagree Neutral Agree	54.3 25.2 20.4	52.4 28.6 18.9	57.5 23.0 19.5	53.2 24.2 22.6	0.176
Canned foods are minimally processed Disagree Neutral Agree	58.1 35.3 6.5	53.7 40.3 5.9	60.0 34.1 6.0	60.4 32.1 7.5	0.075
High fiber foods can give gas Disagree Neutral Agree	10.0 32.6 57.3	9.0 37.6 53.4	10.9 29.5 59.6	10.2 31.1 58.8	0.099
Takes too long to make dry beans from scratch Disagree Neutral Agree	26.6 49.2 24.2	16.9 60.7 22.4	24.3 47.3 28.3	36.9 40.9 22.2	<0.001
Do not like the taste of beans Disagree Neutral Agree	68.7 10.2 21.1	46.9 12.3 40.7	68.6 12.8 18.6	87.4 6.2 6.4	<0.001

**Table 4:** Pulse-cooking experiences of students enrolled at a Midwestern US university by trivariate pulse type count eaten per month (%; *n*=1433).

Pulse Cooking Variables	Total	No Pulses Eaten 32% (452)	1–2 Types Eaten 31% (451)	3-11 Types Eaten 37% (530)	*p*-Value
	%				
Knows how to cook dry pulses Yes No/Do not know	37.2 62.8	23.9 76.1	35.3 64.7	50.2 49.8	<0.001
Experience in cooking dry pulses Yes, recently in past 4 weeks Yes, done in past No	12.4 23.6 64.1	6.4 16.2 77.4	8.0 26.2 65.9	21.1 27.7 51.1	<0.001
**Of the 36% (515/1433) who have ever cooked pulses**					
Experience described was first time Yes No	29.4 70.6	39.6 60.4	32.97 67.3	23.3 76.7	0.006
Most recent pulses cooked Black beans Lentils Pinto beans Chickpeas Split peas Navy, kidney, other Black eyed peas, mung beans Do not remember	24.0 23.8 12.5 12.3 6.4 5.1 4.9 11.1	18.8 17.8 14.9 14.9 6.9 2.0 6.9 17.8	20.8 24.7 16.9 8.4 7.1 5.8 5.2 11.0	27.9 25.6 8.9 13.6 5.8 5.8 3.9 8.5	0.055
Soaked prior to cooking Yes No No, type did not need soaking Do not remember	66.5 11.1 11.9 10.5	67.3 12.9 10.9 8.9	66.9 9.7 12.3 11.0	65.9 11.2 12.0 10.9	0.985
Cooking method used Pot on a stove Pressure Slow cooker Do not remember	66.5 16.9 10.0 6.7	61.4 14.9 12.9 10.9	69.9 13.1 9.8 7.2	66.4 19.9 9.0 4.7	0.173
Quality of pulses cooked Pulses were good to eat Pulses were not good to eat	87.5 12.5	80.2 19.8	85.7 14.3	91.4 8.6	0.011
**Of the 13% (64/515) who said pulses were not good**					
Did not like taste of this type Preparation problems	31.3 68.8	45.0 55.0	77.3 22.7	81.8 18.2	0.021

**Table 5:** Logistic regression model of predictors of pulse type consumption of students enrolled at a Midwestern US university (*n* = 1433).

Variables in the Regression Model			95% Confidence Interval for Odds Ratio		
	Beta (SE)	Significance	Lower	Odds Ratio	Upper
Cooking self-efficacy	0.052 (0.015)	<0.001	1.023	1.053	1.084
Only poor people eat beans	−0.280 (0.094)	0.003	0.629	0.756	0.908
I do not like the taste of beans	−0.626 (0.051)	<0.001	0.484	0.535	0.591
White or Other Races (1)	0.341 (0.161)	0.034	1.025	1.407	1.930
Vegetarian/vegan (1)	0.551 (0.276)	0.046	1.009	1.735	2.982
Constant	1.221 (0.419)	0.004		3.392	
Percent correct	No pulse types eaten often		37.8	Overall	73.2
	1 or more pulse type eaten often		89.4		
Model significance	*p* < 0.001				

## References

[1] U.S. Department of Health and Human Services, U.S. Department of Agriculture (2015). Dietary Guidelines for Americans 2015.

[2] Winpenny E.M., Van Sluijs E.M., White M., Klepp K.-I., Wold B., Lien N. (2018). Changes in diet through adolescence and early adulthood: longitudinal trajectories and association with key life transitions. Int. J. Behav. Nutr. Phys. Act..

[3] Stok F.M., Renner B., Clarys P., Lien N., Lakerveld J., Deliens T. (2018). Understanding Eating Behavior during the Transition from Adolescence to Young Adulthood: A Literature Review and Perspective on Future Research Directions. Nutrients.

[4] Harris D.A. (2017). Just the “Typical College Diet”: How College Students Use Life Stages to Account for Unhealthy Eating. Symb. Interact..

[5] Farhat G., Lees E., Macdonald-Clarke C., Amirabdollahian F. (2019). Inadequacies of micronutrient intake in normal weight and overweight young adults aged 18–25 years: A cross-sectional study. Public Health.

[6] American College Health Association (2020). American College Health Association-National College Health Assessment III: Reference Group Executive Summary Spring 2020.

[7] Richards R., Brown L.B., Williams D.P., Eggett D.L. (2017). Developing a Questionnaire to Evaluate College Students’ Knowledge, Attitude, Behavior, Self-efficacy, and Environmental Factors Related to Canned Foods. J. Nutr. Educ. Behav..

[8] Drury R.H., Brown L.B., Williams P., Eggett D., Richards R. (2018). College Students’ Understandings of, Perceptions Towards, and Usage of Canned Foods Based on Exposure to Canned Foods During Childhood. https://scholarsarchive.byu.edu/studentpub_uht/28.

[9] Clark S., Duncan A.M. (2017). The role of pulses in satiety, food intake and body weight management. J. Funct. Foods.

[10] Food and Agriculture Organization (1994). Definition and Classification of Commodities. http://www.fao.org/waicent/faoinfo/economic/faodef/fdef04e.htm.

[11] Mitchell D.C., Lawrence F.R., Hartman T.J., Curran J.M. (2009). Consumption of Dry Beans, Peas, and Lentils Could Improve Diet Quality in the US Population. J. Am. Diet. Assoc..

[12] Havemeier S., Erickson J., Slavin J.L. (2017). Dietary guidance for pulses: the challenge and opportunity to be part of both the vegetable and protein food groups. Ann. N. Y. Acad. Sci..

[13] Messina V. (2014). Nutritional and health benefits of dried beans. Am. J. Clin. Nutr..

[14] Freed A., Wong D. (2019). The relationship between university students’ environmental identity, decision-making process, and behavior. J. Sustain. Educ..

[15] Pelletier J.E., Laska M.N., Neumark-Sztainer D., Story M. (2013). Positive attitudes toward organic, local, and sustainable foods are associated with higher dietary quality among young adults. J. Acad. Nutr. Diet..

[16] Foyer C.H., Lam H.-M., Nguyen H.T., Siddique K.H.M., Varshney R.K., Colmer T.D., Cowling W., Bramley H., Mori T.A., Hodgson J.M. (2016). Neglecting legumes has compromised human health and sustainable food production. Nat. Plants.

[17] Willett W., Rockström J., Loken B., Springmann M., Lang T., Vermeulen S., Garnett T., Tilman D., Declerck F., Wood A. (2019). Food in the Anthropocene: The EAT-Lancet Commission on healthy diets from sustainable food systems. Lancet.

[18] Sowers M.F., Colby S.E., Allison C.L., Zhou W. (2018). Development and Validation of a BEAN Survey for College Students. Food Nutr. Sci..

[19] Masuda K. (2018). Pulses as Culturally Important Foods among University Students in Canada. J. Food Res..

[20] Hiller M.B. (2019). Food Security and Dietary Acculturation among College Students at Iowa State University. Master’s Thesis.

[21] Leiner D.J. (2013). Too Fast, Too Straight, Too Weird: Post Hoc Identification of Meaningless Data in Internet Surveys. SSRN Electron. J..

[22] Martinez S.M., Webb K., Frongillo E.A., Ritchie L.D. (2018). Food insecurity in California’s public university system: What are the risk factors?. J. Hunger. Environ. Nutr..

[23] Block G., Gillespie C., Rosenbaum E.H., Jenson C. (2000). A rapid food screener to assess fat and fruit and vegetable intake. Am. J. Prev. Med..

[24] NutritionQuest Web-Based Wellness Solutions Free Assessment Tools for Individuals. https://nutritionquest.com/wellness/free-assessment-tools-for-individuals/.

[25] United States Department of Agriculture, Economic Research Service U.S. Household Food Security Survey Module: Six-Item Short Form. https://www.ers.usda.gov/media/8282/short2012.pdf.

[26] Winham D.M., Hutchins A.M., Thompson S.V., Dougherty M.K. (2018). Arizona Registered Dietitians Show Gaps in Knowledge of Bean Health Benefits. Nutrients.

[27] Winham D.M., Florian T.A. (2010). Hispanic Women in EFNEP Have Low Adherence With Dietary Guidelines Regardless of Acculturation Level. J. Hunger. Environ. Nutr..

[28] Winham D.M., Tisue M.E., Palmer S.M., Cichy K.A., Shelley M.C. (2019). Dry Bean Preferences and Attitudes among Midwest Hispanic and Non-Hispanic White Women. Nutrients.

[29] Lucier G., Lin B.H., Allshouse J., Kantor L.S. (2000). Factors affecting dry bean consumption in the United States. Veg. Spec. Situat. Outlook.

[30] Global Pulse Confederation Pulses Cooking Times: Citizen Science. https://pulses.org/pulses-cooking-times.

[31] Iowa State University Report. https://www.registrar.iastate.edu/resources/enrollment-statistics/sex-ethnicity-residence-reports.

[32] Segovia-Siapco G., Sabaté J. (2019). Correction: Health and sustainability outcomes of vegetarian dietary patterns: a revisit of the EPIC-Oxford and the Adventist Health Study-2 cohorts. Eur. J. Clin. Nutr..

[33] Bowman S.A. (2020). A Vegetarian-Style Dietary Pattern Is Associated with Lower Energy, Saturated Fat, and Sodium Intakes; and Higher Whole Grains, Legumes, Nuts, and Soy Intakes by Adults: National Health and Nutrition Examination Surveys 2013–2016. Nutrients.

[34] Larson N.I., Perry C.L., Story M., Neumark-Sztainer D. (2006). Food Preparation by Young Adults Is Associated with Better Diet Quality. J. Am. Diet. Assoc..

[35] Papanikolaou Y., Fulgoni V.L. (2008). Bean consumption is associated with greater nutrient intake, reduced systolic blood pressure, lower body weight, and a smaller waist circumference in adults: results from the National Health and Nutrition Examination Survey 1999–2002. J. Am. Coll. Nutr..

[36] Heer M.M., Winham D.M. (2020). Bean Preferences Vary by Acculturation Level among Latinas and by Ethnicity with Non-Hispanic White Women. Int. J. Environ. Res. Public Health.

[37] Heer M.M., Winham D.M. (2020). Food Behaviors, Health, and Bean Nutrition Awareness among Low-Income Men: A Pilot Study. Int. J. Environ. Res. Public Health.

[38] Radford A., Dahl W.J. (2014). Identifying learning needs of WIC participants regarding dry beans. JNEAFCS.

[39] Doma K.M., Farrell E.L., Leith-Bailey E.R., Soucier V.D., Duncan A.M. (2019). Motivators, Barriers and Other Factors Related to Bean Consumption in Older Adults. J. Nutr. Gerontol. Geriatr..

[40] Szczebyło A., Rejman K., Halicka E., Laskowski W. (2020). Towards More Sustainable Diets—Attitudes, Opportunities and Barriers to Fostering Pulse Consumption in Polish Cities. Nutrients.

[41] Winham D.M., Hutchins A.M. (2011). Perceptions of flatulence from bean consumption among adults in 3 feeding studies. Nutr. J..

[42] Palmer S.M., Winham D.M., Hradek C. (2018). Knowledge gaps of the health benefits of beans among low-income women. Am. J. Health Behav..

[43] Jones S.A., Walter J., Soliah L., Phifer J.T. (2014). Perceived Motivators to Home Food Preparation: Focus Group Findings. J. Acad. Nutr. Diet..

[44] Murray D.W., Mahadevan M., Gatto K., O’Connor K., Fissinger A., Bailey D., Cassara E. (2015). Culinary efficacy: An exploratory study of skills, confidence, and healthy cooking competencies among university students. Perspect. Public Health.

[45] Comerford K.B. (2015). Frequent Canned Food Use is Positively Associated with Nutrient-Dense Food Group Consumption and Higher Nutrient Intakes in US Children and Adults. Nutrients.

[46] Bassett A., Dolan K.D., Cichy K. (2020). Reduced retort processing time improves canning quality of fast-cooking dry beans (*Phaseolus vulgaris* L.). J. Sci. Food Agric..

[47] Irazusta A., Hoyos I., Irazusta J., Ruiz F., Diaz E., Gil J. (2007). Increased cardiovascular risk associated with poor nutritional habits in first-year university students. Nutr. Res..

[48] Niva M., Vainio A., Jallinoja P. (2017). Barriers to Increasing Pulse Consumption in Western Populations. Vegetarian and Plant-Based Diets in Health and Disease Prevention.

[49] Melendrez-Ruiz J., Buatois Q., Chambaron S., Monnery-Patris S., Arvisenet G. (2019). French consumers know the benefits of pulses, but do not choose them: An exploratory study combining indirect and direct approaches. Appetite.

[50] Figueira N., Curtain F., Beck E.J., Grafenauer S. (2019). Consumer Understanding and Culinary Use of Legumes in Australia. Nutrients.

[51] Higgins C. Perfect Timing: Creating a Database of Pulse Cooking Times, One Pot at a Time. https://pulsepod.globalpulses.com/research-corner/post/perfect-timing-creating-a-database-of-pulse-cooking-times-one-pot-at-a-time.

